# Transcriptome- and DNA methylation-based cell-type deconvolutions produce similar estimates of differential gene expression and differential methylation

**DOI:** 10.1186/s13040-024-00374-0

**Published:** 2024-07-11

**Authors:** Emily R. Hannon, Carmen J. Marsit, Arlene E. Dent, Paula Embury, Sidney Ogolla, David Midem, Scott M. Williams, James W. Kazura

**Affiliations:** 1 Center for Global Health and Diseases, Case Western Reserve University, 10900 Euclid Avenue LC:4983, Cleveland, OH 44106 USA; 2 Department of Population and Quantitative Health Sciences, Case Western Reserve University, Cleveland, OH 44106 USA; 3 Gangarosa Department of Environmental Health, Rollins School of Public Health, Emory University, Atlanta, GA 30322 USA; 4 Division of Pediatric Infectious Diseases, Rainbow Babies and Children’s Hospital, Cleveland, OH 44106 USA; 5 Kenya Medical Research Institute, Kisumu, Kenya; 6 Chulaimbo Sub-county Hospital, Kisumu County, Kenya; 7 Department of Population and Quantitative Health Sciences, Cleveland Institute for Computational Biology, Case Western Reserve University, Cleveland, OH 44106 USA

**Keywords:** Deconvolution, PBMC, Gene expression

## Abstract

**Background:**

Changing cell-type proportions can confound studies of differential gene expression or DNA methylation (DNAm) from peripheral blood mononuclear cells (PBMCs). We examined how cell-type proportions derived from the transcriptome versus the methylome (DNAm) influence estimates of differentially expressed genes (DEGs) and differentially methylated positions (DMPs).

**Methods:**

Transcriptome and DNAm data were obtained from PBMC RNA and DNA of Kenyan children (*n* = 8) before, during, and 6 weeks following uncomplicated malaria. DEGs and DMPs between time points were detected using cell-type adjusted modeling with Cibersortx or IDOL, respectively.

**Results:**

Most major cell types and principal components had moderate to high correlation between the two deconvolution methods (*r* = 0.60–0.96). Estimates of cell-type proportions and DEGs or DMPs were largely unaffected by the method, with the greatest discrepancy in the estimation of neutrophils.

**Conclusion:**

Variation in cell-type proportions is captured similarly by both transcriptomic and methylome deconvolution methods for most major cell types.

**Supplementary Information:**

The online version contains supplementary material available at 10.1186/s13040-024-00374-0.

## Background

Human peripheral blood mononuclear cells (PBMCs) are commonly studied in immunology because they contain both innate and adaptive immune cells, are relatively easy and inexpensive to collect, and are readily preserved in biobanks. Interrogation of PBMCs offers a snap shot of the immune response with respect to epigenetic regulation of gene expression since many critical cell types are captured simultaneously. In contrast, studies with purified cell subsets are easier to interpret, but do not capture the overall immune response and require extensive laboratory resources to isolate cell subsets well. For example, bulk transcriptional signatures from antigen-stimulated PBMCs before and after live malaria sporozoite immunization can predict protection against subsequent experimental malaria challenge [1]. A trade-off in using PBMCs for epigenetic and gene expression studies is that information about cell-type proportions is necessary to interpret the source of differential responses, making the understanding of studies with heterogeneous cell populations challenging [2–5].

Cell-type composition can act as a confounder, mediator, precision variable, or biomarker of immune response analyses [2, 5, 6]. Each cell type is characterized by a unique gene expression and DNA methylation profile. Therefore, when cell-type heterogeneity is present, such as in peripheral blood, gene expression and DNA methylation profiles can be largely driven by the cell types present [2, 3]. For example, variation in cell-type proportions was identified as a confounding factor in the analysis of differential gene expression of placental tissues between preeclampsia cases and controls that caused false positive associations [2].

Cell-type heterogeneity can be addressed by different study designs. These include single-cell sequencing, negative or positive isolation of cell subsets, or cell-type adjusted modeling strategies of bulk samples [6]. These methods vary greatly in feasibility, cost, and biases. Single-cell technology remains inaccessible in many contexts due to its high cost when large sample sizes are required. Additionally, depth of coverage per gene in a single cell is often low, resulting in gene expression profiles that are subject to high levels of noise and missing data [7]. Alternatively, PBMCs can be purified into various cell subsets prior to analysis. Both of these methods are constrained by sample quantity, involve extra steps in processing that can alter cellular activation states, and do not fully eliminate heterogeneity [8]. Lastly, cell proportions can be estimated from bulk gene expression or DNA methylation using statistical inference approaches. There are many reference-based and reference-free deconvolution methods that estimate cell-type composition from RNA sequencing data, but fewer exist for use with epigenetic data [9–12].

Cell proportion estimates produced by deconvolution methods generally correlate well with estimates measured by flow cytometry [10]. However, validation and generalization of these deconvolution algorithms and reference matrices is inherently challenging. Artificial nucleic acid mixtures, computer- simulated mixtures, and/or flow cytometry are used as ground truth measurements to assess quality of cell-type proportion estimates under the assumption that all the cell types are represented [10, 11, 13]. It is questionable how well reference-based cell proportion estimates perform across diverse human populations, age ranges, or immune states that are not represented in the validation comparisons. Current evidence based on some deconvolution methods [14] indicates less accurate estimates in females than males, older individuals, smokers, and neonates.

In the analysis described here, whole transcriptome RNA sequencing data and genome-wide DNA methylation data were collected from the same PBMC samples and independently used to infer cell-type proportions in Kenyan children before, during, and following uncomplicated febrile malaria. We addressed two major issues relevant to cell-type heterogeneity: (1) how estimated cell-type proportions derived from two independent deconvolution methods correlate with each other, and (2) how cell proportion estimates derived from transcriptome data versus DNA methylation (DNAm) data influence the detection of differentially expressed genes and differentially methylated positions over time.

## Materials and methods

### Peripheral blood mononuclear cell collection and processing

PBMC were isolated from anticoagulated whole blood of Kenyan children by hypaque ficoll density gradient centrifugation and cryopreserved at baseline before febrile malaria (“baseline” time point), during uncomplicated febrile malaria immediately preceding administration antimalarial drugs (“malaria” time point), and 6 weeks after recovery (“recovery” time point) as described previously [15]. An exploratory subset of 24 samples from 8 children were examined. All children had non-life-threatening uncomplicated malaria defined by fever (temp > 37.9°) and a positive blood smear for *Plasmodium falciparum* (Pf) parasites. The study participants were all of Luo ethnicity, 5–8 years old, and included 3 males and 5 females. All children had negative blood smears for Pf and other *Plasmodium* species at baseline and recovery that were retrospectively confirmed negative by PCR for Pf 18 S ribosomal RNA gene. Cryopreserved PBMCs were thawed using the 10X genomics protocol (Fresh Frozen Human Peripheral Blood Mononuclear Cells for Single Cell RNA sequencing CG00039 revD, 10X Genomics). The studies were approved by the IRB of the Kenya Medical Research Institute (SSC Protocol 2207) and University Hospitals Cleveland Medical Center/Case Western Reserve University (#06-11-22 and #20,190,666).

**Fig. 1:**
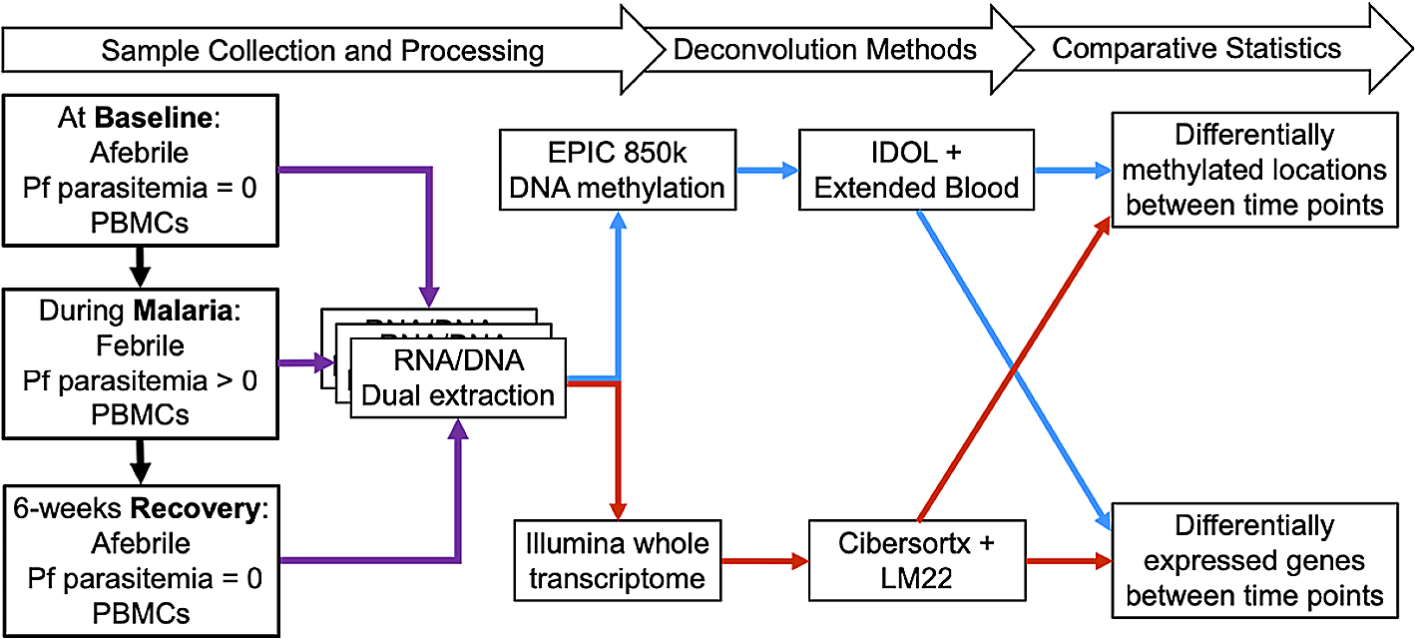
Steps for data collection and analyses. Purple lines go from the raw samples to the co-extraction step. Blue lines represent DNA-methylation-derived deconvolution methods and red lines represent transcriptome-derived deconvolution methods. For RNA, steps included Illumina whole transcriptome followed by deconvolution with the Cibersortx and the LM22 reference. For DNA, the EPIC chip was used to measure the DNA methylation across 850 K probe sites and then used for deconvolution with the IDOL and the extended blood reference [11]. The results from each deconvolution were used as covariates to model the differential gene expression and differential methylation represented by the crossing red and blue lines going from deconvolution method to differential gene expression and DNA methylation

RNA and DNA were extracted from the same PBMC sample from each individual at each time point using the Allprep DNA/RNA mini kit (QIAGEN^®^). RNA and DNA quality were assessed with Agilent TapeStation ScreenTapes and quantified by Qubit assay prior to analyses. Whole genome RNA sequencing was obtained from paired, stranded 100 bp read-lengths on an Illumina platform performed at the Cleveland Clinic Genomics Core. DNAm data were obtained with the Infinium Methylation EPIC BeadChip Kit. EPIC assays were run at the Case Western Reserve University School of Medicine Genomics core facility. These combined assays consumed the entire nucleic acid sample due to small sample volumes from this pediatric population. Figure 1 depicts the general methods followed in this analysis.

### Data processing

Transcriptomics: After visual inspection of fastQC plots [16], gene counts were calculated from raw fastq files using STAR for alignment and HTSeq for gene counts [17, 18]. All annotations and reference genomes used for gene-count calculations were obtained from GENCODE [19].

DNA methylation: The Minfi package in R was used to complete quality control, normalization with FunNorm, BMIQ adjustment for probe-type, filtering off-target probes, and filtering of age-associated probes [20–22]. A batch correction was not applied to correct for slide-level differences since this could introduce bias given the slide design [23].

### Deconvolution methods

Transcriptome: Transcriptome derived cell-type proportions were estimated using Cibersortx [13] and the LM22 reference panel [24] with B-mode batch correction in relative mode. LM22 was originally generated from previously published microarrays of purified cell subsets and included 22 immune cell types. The Cibersortx B-mode corrects for cross-platform variation and extends the utility of LM22 to RNA-seq data [13]. LM22 has been validated for use in PBMCs [24].

Differences in cell-type proportion between time points were detected using the nonparametric paired Friedman test followed by Wilcoxon signed-rank tests with Bonferroni correction for multiple testing to obtain contrast-level resolution [25]. Nonparametric tests were used to account for the non-normal behavior of proportional data.

DNA methylation: DNA methylation (DNAm) derived cell-type proportions were calculated using methods with the extended blood reference as previously described [11]. The reference panel was made with EPIC chip DNA methylation profiles of purified cell subsets from a mixed population and includes 12 immune cell types present in whole blood. Differences in cell-type proportion between time points were detected as described above for RNA-seq data.

### Detecting differential gene expression and methylation between baseline, febrile malaria, and recovery

Unadjusted or cell-type adjusted linear mixed models were created to detect differentially expressed genes (DEGs) or differentially methylated positions (DMPs) between baseline, malaria, and recovery with the limma package in R [26, 27]. Subject level gene expression or DNA methylation variation was accounted for by including a random effect [28]. For transcriptomic data, the limma-voom [29] transformation was used to adjust for the non-normality of gene count data. For the DNA methylation data, a logit transformation was applied to convert beta values that are bound by 0 and 1 to m-values [30].

Two approaches of adjusting for cell-type composition were used: (1) adding each estimated cell proportion as a covariate or (2) adding the first two principal components of cell-type proportions as covariates. In the first approach, 5 separate models with adjustments were constructed for only monocytes, naïve B cells, naïve CD4 T cells, CD8 T cells, or memory CD4 T cells to avoid overfitting. The second approach was included to capture a multidimensional cell-type adjustment with the small sample size. Centered and un-scaled principal components were calculated from the transcriptome-derived or the DNA-methylation-derived matrix of cell-type proportion estimates using the singular value decomposition algorithm in the prcomp() function in R following methods previously described [2]. For each cell-type adjusted model, corresponding models were created using the estimates from the transcriptome or DNA methylation array, respectively.

### Comparative statistics

Between cell-type proportion estimates: Pearson correlations between cell-type proportion estimates from the two deconvolution methods were calculated for each cell subset. Linear regressions were fit to describe the shape of the association between deconvolution methods. Owing to differences between cell types represented in reference matrices, some estimated proportions were added together to match the cellular categorization when necessary (Supplementary Table S1). The differences between reference matrices are: the LM22 reference divided the CD4 memory T cells into activated and resting while the extended blood reference only estimated total CD4 memory T cells; CD8 T cells were divided into naïve and memory subsets in the extended blood reference and estimated as total CD8 T cells in LM22; NK cells were divided into activated and resting in the LM22 reference but only estimated as total NK cells in the extended blood reference. Additionally, the first and second principal components derived from each deconvolution approach were tested for correlation with Pearson method.

Differential gene expression and methylation analyses: The number of DEGs or DMPs was calculated at multiple P-value thresholds for each model and compared across models. Additionally, the logFC for gene expression or DNA methylation CpG locations were compared between deconvolution approaches for the genes with the 100 smallest p-values in any of the models between any of the 3 time points: baseline; during malaria; and recovery. These comparisons resulted in 779 high-interest genes (supplementary dataset 1). Similarly, 1356 high-interest CpG locations were identified as those with the 100 smallest p-values among any of the 3 time points or any of the differential methylation models (supplementary dataset 2). Genes or CpG locations were considered “deconvolution-sensitive” when the orthogonal distance between the estimated point and the 1:1 line was in the lower 0.15 percentile or higher than the 99.85 percentile, which represents extreme events in the dataset. These percentile values are equivalent to those more than 3 standard deviations (sd) from a normally distributed variable. The distribution of the orthogonal distances is over-dispersed and follows a Laplace’s distribution with µ = 0 and b = sd/2, meaning that the sd measurement is an underestimate of true variance. Distribution percentiles were therefore used to select deconvolution sensitivity.

## Results

### Deconvolution estimates and changes in cell-type composition at baseline, during malaria, and at recovery

**Fig. 2:**
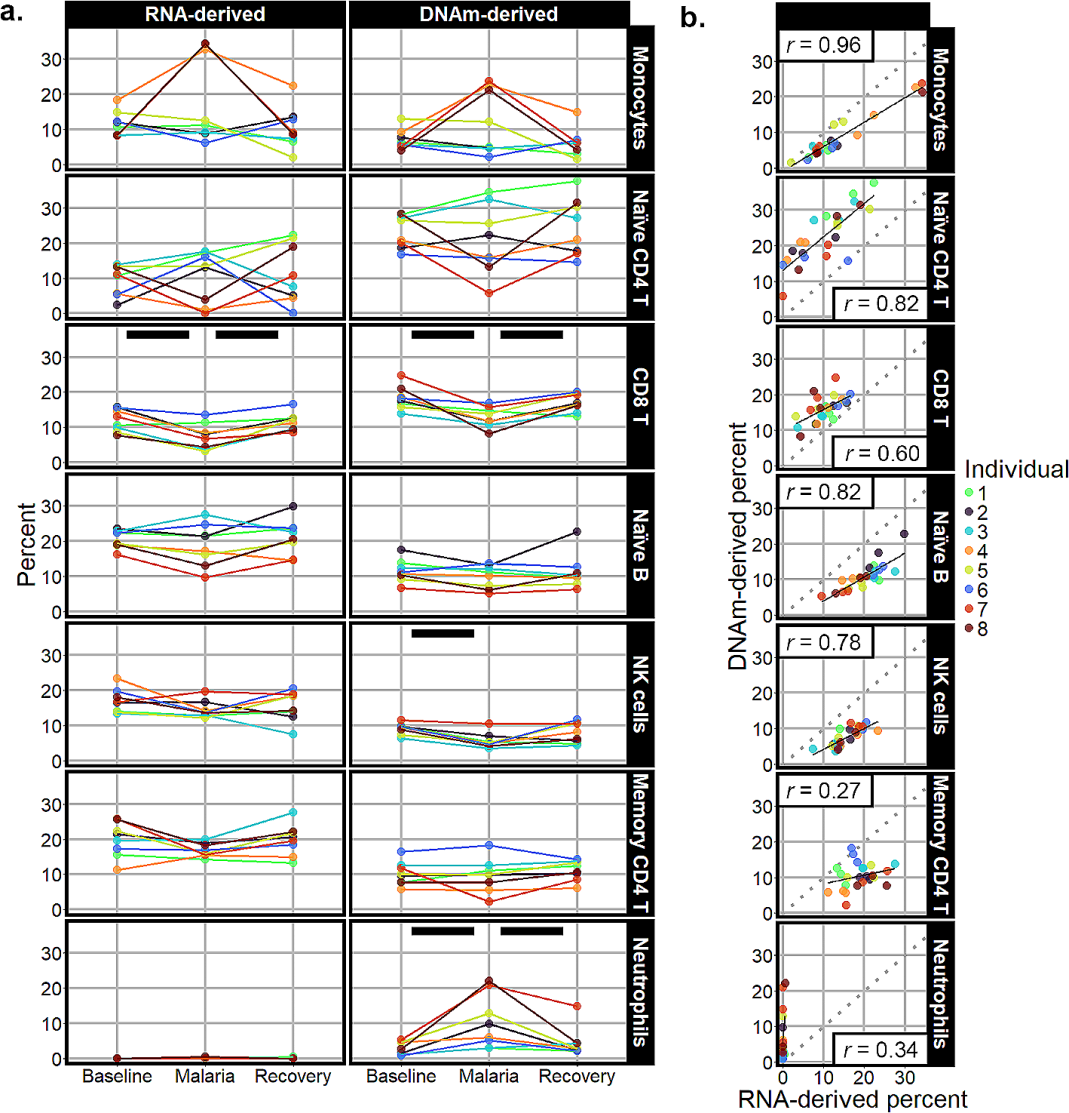
**a**. Cell percent estimates derived from the transcriptome (column labeled “RNA-derived”) and DNA methylome (column labeled “DNAm-derived”). Black bars indicate a statistically significant change in cell-type proportion. **b**. Scatter plots and linear model fits of association between cell proportion estimates from transcriptome-derived methods and DNAm-derived methods. The Pearson correlation value *r* is labeled in correlation plots. Each individual is labeled a different color

Transcriptome-derived deconvolution estimates detected three statistically significant changes in cell-type proportions across time points (Fig. 2a, Supplementary Table S2). First, CD8 T cell proportions differed by timepoint (p-value = 0.01). At baseline, CD8 T cells averaged 11.96% of cells, dropped to 7.47% during malaria, and returned to 11.61% at recovery. Similarly, memory resting CD4 T cells showed a drop in cell proportion during malaria from 19.09% at baseline to 13.85%, and then returned to 19.28% at recovery (p-value = 0.03). Lastly, in spite of overall low proportions, there was a statistically significant increase in M0 macrophages during malaria, starting at 0.03%, increasing to 0.33%, and returning to 0.01% (p-value = 0.02). Monocytes, natural killer cells, naïve B cells, and naïve CD4 T cells showed no detectable changes; all were estimated to be between 10% and 33%. The remainder of the cell types included in LM22 estimated proportions between 0% and 4.33% (Supplementary Figure S1).

DNA-methylation-derived deconvolution estimates (Fig. 2a, Supplementary Table S3) also had three cell types with detectable differences across time points. Consistent with the RNA-derived proportions, there was a reduction of the memory CD8 T cells during malaria with the average percent cells dropping from 10.40% at baseline to 4.55% during malaria and returning to 8.51% at recovery (p-value = 0.01). Natural killer cell proportions also differed with the average cell percent dropping from 9.04 to 5.60% at baseline versus malaria and returning to 7.69% at recovery (p-value = 0.01). Lastly, neutrophils had an increase during malaria from baseline measure of 2.78–10.38%, followed by a decrease at recovery to 4.48% (p-value < 0.01). The cell types that had proportions greater than 10%, but did not show significant shifts, were monocytes, naïve B cells, memory CD4 T cells, and naïve CD4 T cells. Cell types with lower average estimates (Supplementary Figure S1) included regulatory T cells (total average across time points 4.00%), memory B cells (total average 6.91%), naïve CD8 T cells (total average 8.25%), eosinophils (undetected), and basophils (total average 0.24%).

### Correlation between transcriptome-derived and DNAm-derived cell proportion estimates

**Fig. 3:**
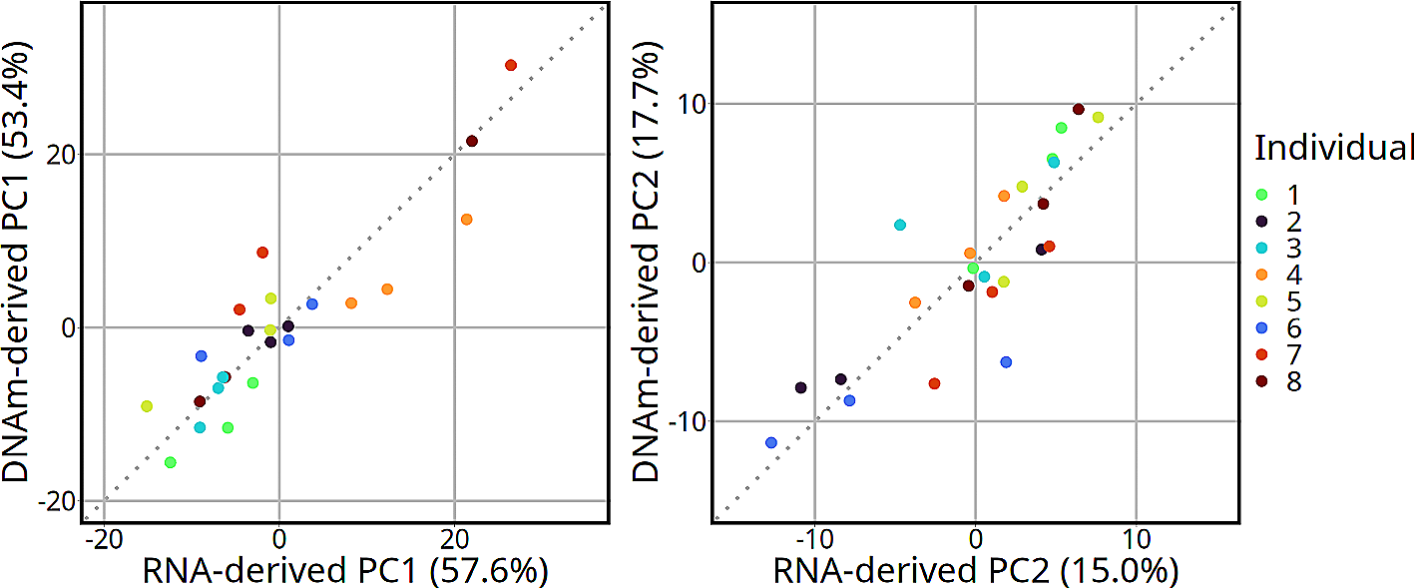
Correlation between the first and second principal components from RNA-derived and DNAm-derived deconvolution methods. Dotted lines represent the identity line. The percent of total variation captured by each respective principal component is in parentheses. The Pearson correlation coefficients left to right were 0.90 and 0.85 (both correlations have a *p*-value < 0.001). The x-axes represent the values for the first (PC1) and second (PC2) that were calculated with the transcriptome-derived deconvolution. The y-axes represent the values for the first (PC1) and second (PC2) from the DNAm-derived deconvolution

Monocytes were the most correlated cell type between the two deconvolution methods with a Pearson correlation coefficient of 0.96 (p-value < 0.001; Fig. 2b, Supplementary Table S4). Other highly correlated cell types were naïve B cells and naïve CD4 T cells, each with a correlation coefficient of 0.82 (p-values < 0.001). Natural killer and CD8 T cell proportions were less correlated with 0.78 and 0.60 coefficients, respectively (p-values < 0.001) The cell-types that were not significantly correlated (p-value > 0.05) were neutrophils, memory CD4 T cells, memory B, regulatory T cells, and eosinophils. The subsets that were estimated as lower in the DNAm-derived deconvolution were monocytes, naïve B cells, and natural killer cells. The cell subsets that had higher DNA-methylation-derived estimated proportions compared to RNA sequencing were naïve CD4 T cells and CD8 T cells. The most striking discrepancy of cell-type percent estimates was for neutrophils, which ranged between 0 and 0.56% in the transcriptome-derived deconvolution compared to 0.9–22.1% in the DNAm-derived proportions. There was a high correlation between the transcriptome-derived and DNAm-derived first and the second principal components (Fig. 3). The Pearson coefficients were 0.90 and 0.85 (p-values < 0.001) for the first and second principal components, respectively. Additionally, the top loading vectors for both transcriptome-derived and DNAm-derived principal components identified monocytes, naïve CD4 T cells, and CD8 T cells as top contributors to cellular variation (Supplementary Figure S2).

### Effect of deconvolution approach on differential gene expression analysis

**Fig. 4:**
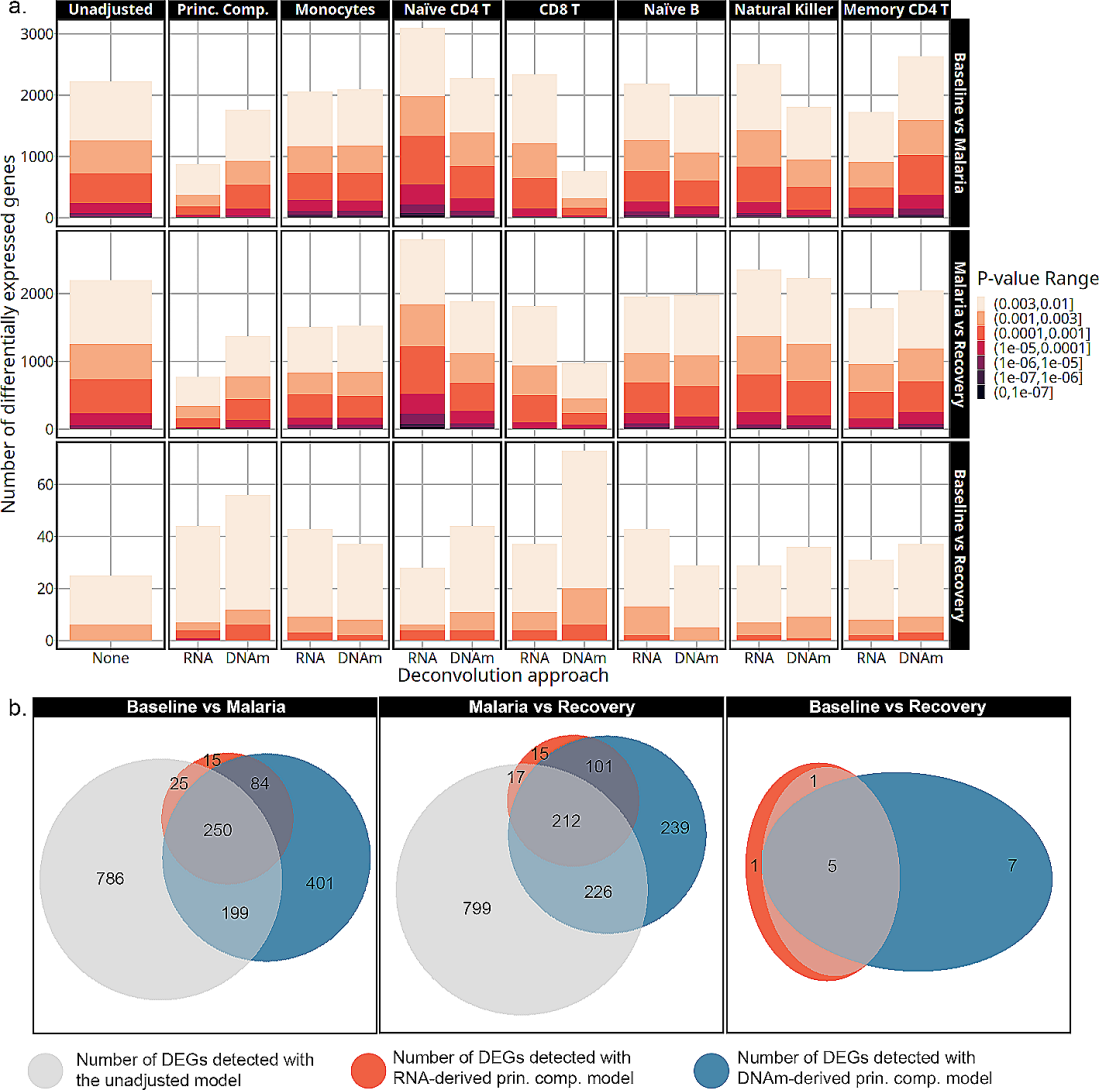
**a**. The number of differentially expressed genes detected by each model is on the y-axis at multiple *p*-value thresholds. The models are indicated by cell-type adjustment labeled across the top, contrast labeled along the right side, and deconvolution approach labeled along the x-axis. Transcriptome-derived and DNAm-derived cell-type adjustments are marked by RNA and DNAm, respectively. As colors become darker, the significance threshold becomes lower. **b**. Venn diagrams showing the overlap in DEGs detected with *p*-value < 0.003 from the unadjusted, transcriptome-derived principal components, and the DNAm-derived principal components models

The number of differentially expressed genes varied by time point, cell-type adjustment, p-value threshold, and deconvolution approach (Fig. 4a). Adjusting differential gene expression for monocytes, naïve CD4 T cells, CD8 T cells, or the principal components resulted in the most variation in the number of DEGs compared to other cell-type adjusted models. Less variation was observed in the number of DEGs detected in models adjusted for naïve B, natural killer, or memory CD4 T cell proportions. Monocyte-adjusted models showed the most consistency across deconvolution approaches, with nearly identical numbers of DEGs between the two methods, an increase in the number of DEGs for the baseline to recovery comparison, and a decrease for the malaria to recovery comparison. Adjusting for transcriptome-derived naïve CD4 T cell proportions resulted in the smallest number of DEGs in the baseline to recovery contrast and the largest number in the baseline to malaria or malaria to recovery compared to any other model. However, this pattern with naïve CD4 T cell adjustment differed from the DNAm-derived adjusted numbers. Lastly, the largest discrepancy in the number of DEGs observed between the deconvolution methods came from adjustment with CD8 T cells. In the baseline to recovery contrast, DNAm-derived adjustment detected double the number of DEGs as the transcriptome-derived correlate. However, there was an increase in the number of DEGs with both adjustment sources relative to the unadjusted model. Conversely, when comparing malaria to the baseline or recovery, there was a dramatic reduction in the detection of DEGs when using the DNAm-derived CD8 T cell estimates that was not observed in the corresponding transcriptome derived model. The multi-cell-type adjusted models that used the top 2 principal components of cell-type proportions as covariates decreased the number of DEGs detected in the baseline to malaria and the malaria to recovery contrasts and increased the number of DEGs in the baseline to recovery contrast with both deconvolution methods.

Genes detected with the principal component adjusted models showed a large overlap between the deconvolution approaches highlighted by the Venn diagrams in Fig. 4b (genes in supplementary dataset 3). Principal component models from both deconvolution approaches had nearly complete overlap with ~ 90% of the DEGs detected with the transcriptome-derived model matching those from the DNAm-derived model. The principal component models only shared ~ 50–70% of the DEGs with the unadjusted models in the baseline to malaria or malaria to recovery contrasts. In the baseline to recovery contrast, 100% of the genes detected in the unadjusted model were also found with the principal components models.

**Fig. 5:**
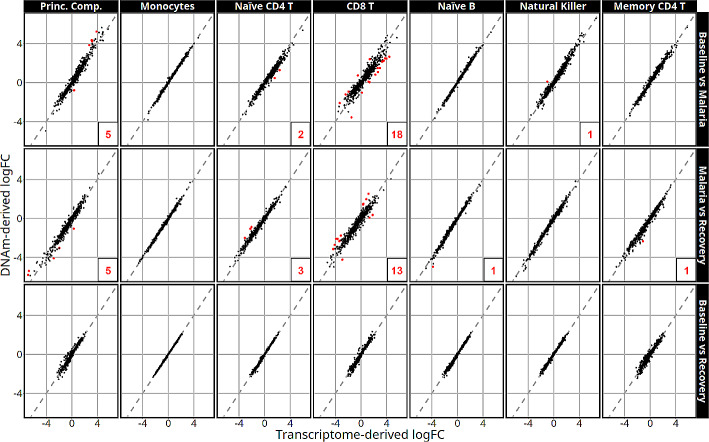
Log fold-change estimates are represented on the x-axis after transcriptome-derived cell-type adjustment versus the corresponding logFC from the DNAm-derived model. Cell-type adjustments are labeled along the top and contrasts along the right side. Red points represent deconvolution-sensitive DEGs (genes whose estimates vary the most using the difference deconvolutions) and their count is listed in the bottom right of each panel if *n* > 0. The dashed line represents the identity line

After cell-type adjustment, the estimated log fold-changes (logFCs) in gene expression between times were similar and follow closely to the identity line. Figure 5 demonstrates the association between the logFC estimates after cell-type adjustment with transcriptome-derived versus DNAm-derived cell proportions in the 779 genes with 100 smallest p values in at least one of the models used. Deconvolution-sensitive genes are labeled in red and are defined as genes with an orthogonal distance between logFC and the identity line in the extreme 0.3 percentile (detailed information in supplementary dataset 4). Estimated logFCs of gene expression were nearly identical in the monocyte, naïve B cell, natural killer, and memory CD4 T cell adjusted models with 0 to 1 deconvolution-sensitive genes found in each cell type (Supplementary Table S5 contains a summary of deconvolution-sensitive DEGs). The highest number of deconvolution-sensitive genes were found within the CD8 T cell adjusted models that identified 18 and 13 of the 779 compared genes in the baseline to malaria and malaria to recovery contrasts, respectively.

### Effect of deconvolution approach on differential methylation analysis

**Fig. 6:**
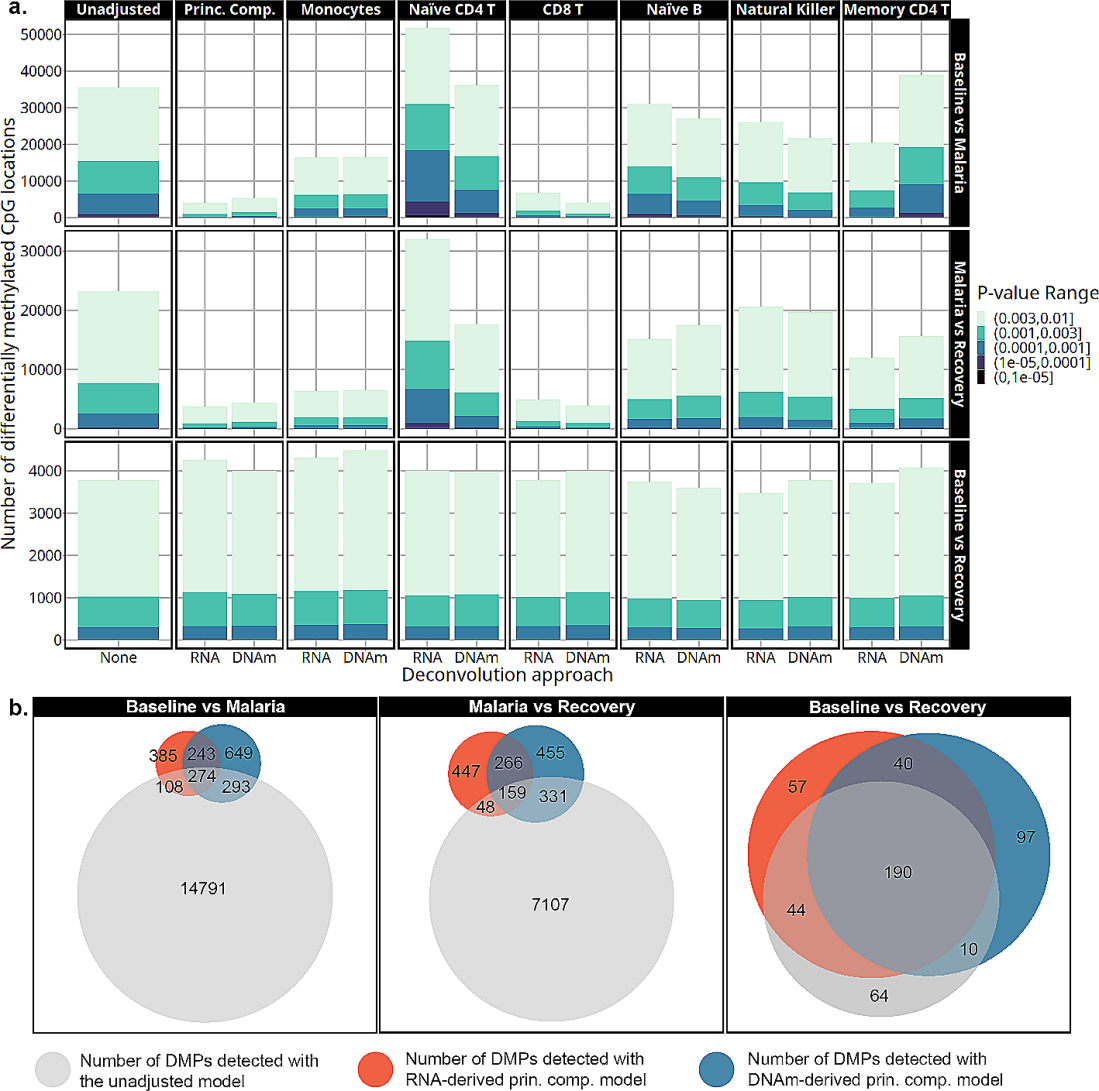
**(a)** The number of differentially methylated positions is on the y-axis at several *p*-value thresholds as detected by cell-type adjustment (labeled across the top), contrast (labeled along the right side), and deconvolution approach (along the x-axis). Transcriptome-derived and DNA-methylation-derived cell-type adjustments are marked by RNA and DNAm, respectively. As colors become darker, the significance threshold becomes higher and are listed in the legend. “Prin. Comp.” refers to the principal component-adjusted models. **(b)** Venn diagrams depict the overlap in DMPs selected with *p*-value < 0.001 in unadjusted, transcriptome-derived principal components, and the DNAm-derived principal components models

The number of differentially methylated positions (DMPs) detected by the cell-type adjusted models varied similarly to the differential gene expression analyses (Fig. 6a). Monocyte, naïve CD4 T cell, CD8 T cell, or principal component adjusted models showed the biggest difference in the number of DMPs compared to the unadjusted model. Smaller differences in the number of DEGs were noted from the models adjusted with naïve B cells, natural killer cells, or memory CD4 T cells. Monocyte adjusted models detected nearly the same number of DMPs across the deconvolution approaches regardless of comparison. Monocyte adjustment resulted in more DMPs in the baseline versus recovery contrast and a reduced number of DMPs detected in the baseline versus malaria and the recovery versus malaria contrasts. Adjustment with only the transcriptome-derived naïve CD4 T cell proportions resulted in the most DMPs compared to any other model in the baseline to malaria and malaria to recovery contrasts. In the baseline to recovery contrast, the number of DMPs did not change after adjustment with naïve CD4 T cell proportions regardless of deconvolution method. Adjustment with CD8 T cell proportions resulted in the most reductions in estimated DMPs with the number decreasing by more than two-thirds when malaria is compared to either baseline or recovery, regardless of the deconvolution method used. Similarly, models that adjusted with principal components also resulted in a large reduction in the number of DMPs when comparing malaria to the other time points. This was observed with both deconvolution methods.

The transcriptome-derived and DNAm-derived principal component models shared most DMPs with the unadjusted model in the baseline versus recovery contrast (Fig. 6b left panel, supplementary dataset 5). The baseline versus malaria or malaria to recovery contrasts were less consistent, where approximately only one-third of the DMPs were shared between the principal component models from the two deconvolution approaches. However, more notable is that models with the principal components adjustment eliminated ~ 90% of the signal detected by the unadjusted models regardless of deconvolution approach in the baseline versus malaria or malaria versus recovery contrasts.

**Fig. 7:**
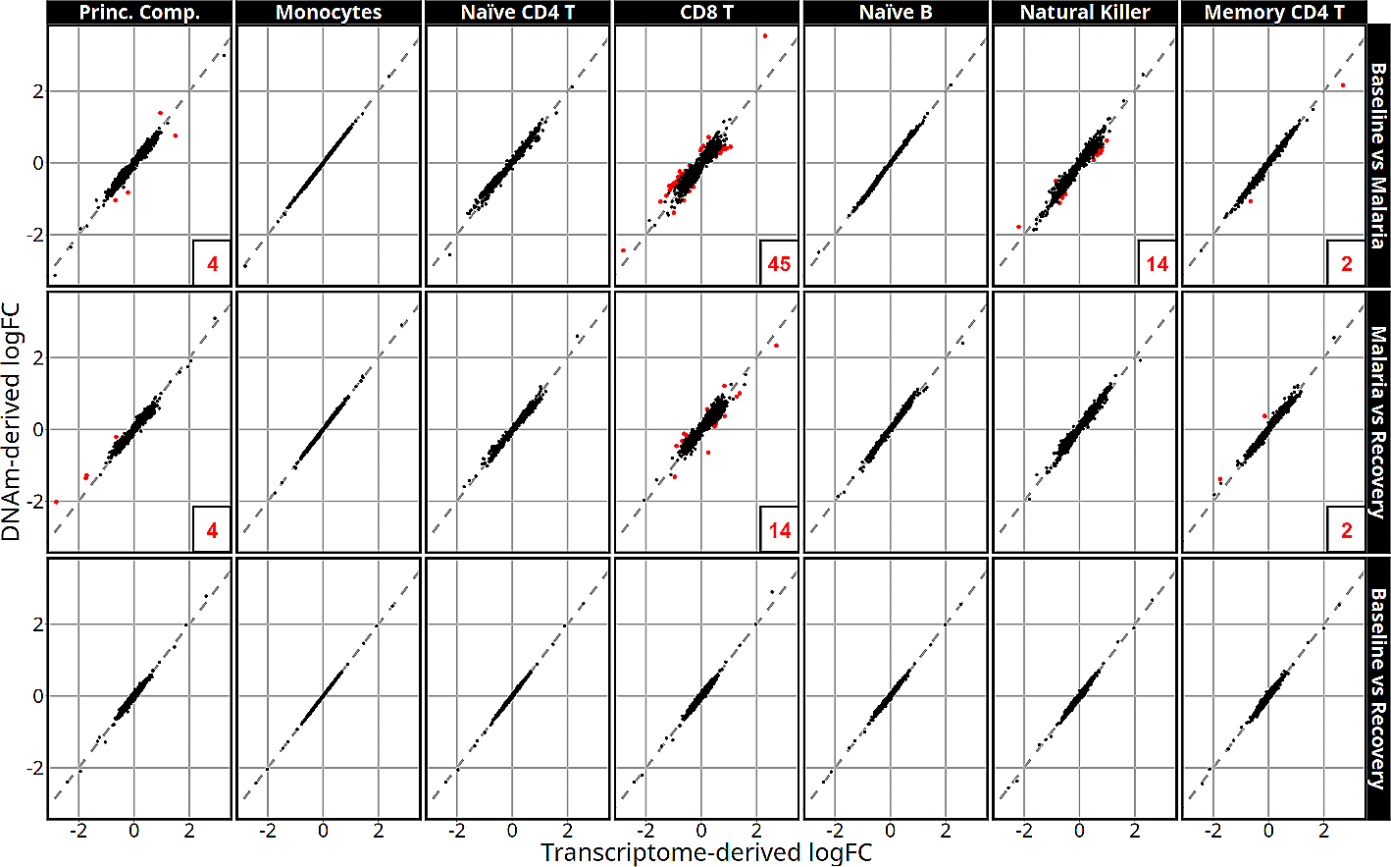
Relative logFC estimates in high interest CpG locations using transcriptome-derived vs. DNAm-derived cell-type adjustments by model (labeled along the top) and contrast (labeled along the right side). Red points are deconvolution-sensitive DMPs and their count is listed in the bottom right corner of each panel if *n* > 0. Dashed lines represent the identity line. Deconvolution-sensitive CpG sites vary the most with different deconvolution approaches

For most cell-type adjustments, the deconvolution approach did not substantially affect the estimated logFC in methylation level between time points as demonstrated by the proximity of the logFC estimates to the identity line (Fig. 7). CpG sites sensitive to deconvolution method are labeled in red on Fig. 7 and are defined as methylation sites with an orthogonal distance between logFC and the identity line in the outer 0.3 percentile (detailed information in supplementary dataset 6). Similar to the differential gene expression analyses, logFCs in monocyte, naïve CD4 T, or naïve B cell-type adjusted models were nearly identical with no deconvolution sensitive DMPs for any contrast in the 1356 CpG sites that have one of the smallest 100 p-values in at least one of the models. Memory CD4 T cells only had 2 deconvolution sensitive genes in the baseline versus malaria and malaria versus recovery contrasts. CD8 T cell adjusted models had the largest discrepancy in logFC between deconvolution methods with 45 and 14 DMPs that were deconvolution-sensitive in the baseline to malaria and recovery to malaria comparisons, respectively (Supplementary Table S6). Unique to the differential methylation analyses, the NK cell adjusted models detected the second most deconvolution-sensitive DMPs with 14 in the baseline to malaria.

## Discussion

Results of our study show that human PBMC cell-type proportion estimates were highly consistent when comparing transcriptome-derived versus DNAm-derived deconvolution methods in a cohort of Kenyan children before, during, and after a febrile malaria illness. Most major cell types that made ~ 10% or greater proportion of the total had correlation coefficients that ranged from *r* = 0.60–0.96. The top two principal components calculated from each deconvolution approach were also highly correlated and identified three cell types, monocytes, CD8 T cells, and naive CD4 T cells, that drive the variation in cell-type proportions in PBMCs. Cell-type adjustments with these same three cell types were associated with the biggest differences in the number of DEGs or DMPs relative to the unadjusted models. Similarly, multi-cell-type adjustment using the top two principal components resulted in the largest change in number of DEGs and DMPs regardless of deconvolution approach. The effect estimates for log-fold change in either differential gene expression or differential methylation in any time point comparisons were largely unaffected by deconvolution method in the cell-type adjusted models. Collectively, these analyses show that both deconvolution methods performed similarly and captured the major sources of cell-type variation despite being based on different nucleic acids, assay platforms, processing pipelines, and reference matrices in a population that differs in age and ethnicity from the deconvolution validation datasets.

During febrile malaria there was a statistically significant decrease in the estimated average CD8 T cell proportions relative to baseline and recovery regardless of the deconvolution method. Adjustment with CD8 T cells substantially reduced the total number of both DEGs and DMPs when comparing malaria to either baseline or recovery samples. Additionally, the most deconvolution-sensitive genes and CpG locations were found in models adjusted for CD8 T cells. This pattern indicates that the decreased CD8 T cell proportions during malaria is confounding the differential gene expression and differential methylation estimates between time points. Reductions in both absolute counts and proportions of CD8 T cells in peripheral blood measured by flow cytometry has been previously reported in children with acute febrile malaria [31–33]. The confounding effect of the change in CD8 T cell proportions highlights the need to account for this cell type in bulk expression or epigenetic study designs related to infectious diseases such as malaria and perhaps other inflammatory states such as bacterial sepsis and autoimmune diseases.

The greatest discrepancy between the deconvolution approaches was found in the estimation of neutrophil proportions, e.g. ranging from ~ 0.10–10% from the transcriptome-derived and DNAm-derived estimate, respectively. Two proposed sources of this discrepancy could be the presence of neutrophil extracellular traps (NETs) in the PBMC compartment or neutrophil contamination and subsequent degradation of neutrophil subsets through cryopreservation processes. Typically, cryopreserved PBMCs contain few neutrophils because they localize to a different layer following hypaque ficoll centrifugation of anticoagulated whole blood and likely do not remain viable following cryopreservation of PBMCs [34, 35]. During febrile malaria illness, NETs have been implicated as major contributors to innate immune pathogenesis [36–38]. Neutrophils release NETs that are composed of cell-free DNA along with other antimicrobial factors to activate innate immune system pathways [39, 40]. NETs could remain in the PBMC compartment during hypaque-ficoll PBMC purification and be detected by the DNA methylation assay. Hence, we speculate that substantially different estimates in neutrophil proportions can in part be explained by the underlying biological differences between analyzing RNA versus DNA in PBMCs.

The two main cell-type adjusted modelling approaches used either a single cell subset or principal components as covariate(s). The value of each approach depends on the intent of the study and features of the system. If the research question is focused on a specific cell type or there is prior knowledge regarding a specific cell type, a single type adjusted model may be most appropriate. Alternatively, if the research interest focuses on global changes in gene expression or differential methylation, then the multi-cell type adjustment with principal components would be more applicable. For example, our study showed that CD8 T cell proportions are acting as a confounder of gene expression and DNA methylation during acute malaria. Therefore, CD8 T cell proportions should be included in the model as either a single covariate or as part of the multiple cell-type model. The use of the multiple cell-type adjusted models resulted in a large loss of significant DEGs and DMPs that may show the degree to which cell-type variation drives gene expression and DNA methylation patterns. On the other hand, the decrease in DEGs and DMPs may be too extreme, thereby eliminating part of the target signal.

There are limitations to the methods used and the results presented here despite the overall consistency between these two deconvolution approaches. In some cell-types, namely memory B cells, regulatory T cells, and memory CD4 T cell subsets, estimates did not correlate between deconvolution approaches. Memory B cells and regulatory T cells are present in small proportions in peripheral blood and harder to detect accurately. The memory CD4 T cells estimates were an exception to this rule but are likely uncorrelated because the transcriptome-derived deconvolution had two sub-categories, activated and resting, whereas the DNAm-derived deconvolution had one total memory CD4 T cells estimate. Nonetheless, cell-type adjusted modeling that accounted for memory CD4 T cells from either deconvolution approach did not substantially affect differential methylation or differential gene expression results. Lastly, the sample size in the current study is small and constrained the assessment of methods available for cell-type adjustment. More cell-type adjusted modelling approaches exist but depend on complex models with many parameters. The challenging logistics surrounding the collection and processing for study designs such as this highly matched, longitudinal study of a pediatric population in rural Africa are reflected in the sample size and are a common problem. Although we cannot determine which method is better when the correlations are poor, the results indicated that, in general, the deconvolution methods do not change the main biological interpretations.

## Conclusions

Cell-type proportion estimates from PBMCs were concordant between transcriptome-derived and DNAm-derived deconvolution approaches in a cohort of Kenyan children before, during, and after a febrile malaria illness with a few exceptions. Furthermore, the estimates for log-fold change in either differential gene expression or differential methylation were similar between deconvolution approaches when applied to cell-type adjusted modelling. Together, these analyses demonstrate the robustness of the deconvolution approaches with the major sources of cell-type variation detected by both deconvolution approaches regardless of different nucleic acids, assay platforms, processing pipelines, and reference matrices.

### Electronic supplementary material

Below is the link to the electronic supplementary material.

Supplementary Material 1

Supplementary Material 2

Supplementary Material 3

Supplementary Material 4

Supplementary Material 5

Supplementary Material 6

Supplementary Material 7

## Data Availability

Data are available in the GEO at https://www.ncbi.nlm.nih.gov/geo/query/acc.cgi?acc=GSE255062.
