# Supplementary material for: Transcriptome- and DNA methylation-based cell-type deconvolutions produce similar estimates of differential gene expression and differential methylation
Source: BioData Min. 2024 Jul 11;17:21. doi: 10.1186/s13040-024-00374-0 (PMC11241886; doi:10.1186/s13040-024-00374-0)
Supplement: Supplementary file 7 — Supplementary Material 7 [file 13040_2024_374_MOESM7_ESM.docx]

**Supplementary Material**

Table of Contents:

|  | **Title** | **Pg** |
| --- | --- | --- |
| *Supplementary Tables* | |  |
| **Table S1** | Scheme for the collapse of cell types to match references | 2 |
| **Table S2** | Differential cell fraction statistics from transcriptome-derived estimates. | 3 |
| **Table S3** | Differential cell fraction statistics from DNA-methylation-derived estimates. | 4 |
| **Table S4** | Between-deconvolution correlation analysis results | 5 |
| **Table S5** | Summary of deconvolution-sensitive DEGs | 6 |
| **Table S6** | Summary of deconvolution-sensitive DMPs | 7 |
|  |  |  |
| *Supplementary Figures* | |  |
| **Figure S1** | Cell proportion estimates for cell types not included on Figure 2a. | 8 |
| **Figure S2** | Loading vectors for top two principal components in transcriptome-derived and DNAm-derived analyses | 9 |

**Supplementary Table S1**  Scheme for the collapse of cell types used to match the reference matrix

| **Estimate used for Comparison** | **Cell Types on LM22** | **Cell Types on Extended Blood** |
| --- | --- | --- |
| Monocyte | Monocyte | Monocyte |
| Natural Killer | Activated natural killer | Natural killer |
|  | Resting natural killer |  |
| Naïve B cell | Naïve B cell | Naïve B cell |
| Memory B cell | Memory B cell | Memory B cell |
| Memory CD4 T | Activated memory CD4 T | Memory CD4 T |
|  | Resting memory CD4 T |  |
| Naïve CD4 T | Naïve CD4 T | Naïve CD4 T |
| CD8 T | CD8 T | Naïve CD8 T |
|  |  | Memory CD8 T |
| Eosinophils | Eosinophils | Eosinophils |
| Neutrophils | Neutrophils | Neutrophils |
| Regulatory T | Regulatory T | Regulatory T |

The cells separated by a dotted line are cell-type proportions that were added together to represent the broader cellular category for between-deconvolution comparisons.

**Supplementary Table S2**  Differential cell fraction statistical test results with Cibersortx and the LM22 reference.

| **Cell Type** | **% at Baseline** | **% at Malaria** | **% at Recovery** | **Friedman P** | **Baseline vs Recovery P** | **Baseline vs Malaria P** | **Malaria vs Recovery P** |
| --- | --- | --- | --- | --- | --- | --- | --- |
| Monocytes | 11.6 | 18.6 | 10.2 | 0.41 | 0.95 | 0.69 | 0.33 |
| **M0 macrophages** | **0.03** | **0.33** | **0.01** | **0.02** | **0.86** | **0.05** | **0.05** |
| M1 macrophages | 0.00 | 0.03 | 0.0 | 0.37 | NA | 1 | 1 |
| M2 macrophages | 0.00 | 0.00 | 0.00 | 0.37 | 1 | NA | 1 |
| Activated NK cells | 0.17 | 0.27 | 0.08 | 0.87 | 1 | 1 | 1 |
| Resting NK cells | 16.75 | 14.19 | 15.44 | 0.42 | 0.55 | 0.33 | 0.55 |
| Memory B cells | 0.36 | 1.45 | 0.39 | 0.81 | 0.79 | 0.63 | 0.63 |
| Naïve B cells | 20.57 | 18.91 | 21.18 | 0.42 | 0.55 | 0.47 | 0.47 |
| Memory activated CD4 T | 0.76 | 3.08 | 0.52 | 0.11 | 0.20 | 0.12 | 0.12 |
| **Memory resting CD4 T** | **19.09** | **13.85** | **19.28** | **0.034** | **1** | **0.06** | **0.05** |
| Naïve CD4 T | 9.49 | 10.31 | 11.32 | 0.88 | 0.95 | 0.95 | 0.95 |
| **CD8 T** | **11.96** | **7.47** | **11.61** | **0.01** | **0.84** | **0.02** | **0.02** |
| Follicular Helper T | 0.34 | 0.10 | 0.40 | 0.35 | 0.86 | 0.63 | 0.60 |
| γδ T cells | 0.00 | 0.00 | 0.00 | NA | NA | NA | NA |
| Regulatory T | 0.46 | 0.29 | 0.93 | 0.30 | 0.67 | 0.68 | 0.62 |
| Activated mast | 3.54 | 4.33 | 4.07 | 0.88 | 1 | 1 | 1 |
| Resting mast | 0.00 | 0.00 | 0.06 | 0.37 | 1 | NA | 1 |
| Activated Dendritic | 0.45 | 1.00 | 0.47 | 0.09 | 0.84 | 0.06 | 0.06 |
| Resting Dendritic | 0.00 | 0.00 | 0.00 | NA | NA | NA | NA |
| Eosinophils | 2.12 | 2.48 | 1.59 | 0.42 | 0.64 | 0.64 | 0.33 |
| Neutrophils | 0.00 | 0.10 | 0.06 | 0.17 | 1 | 0.54 | 0.88 |
| Plasma cells | 2.31 | 3.18 | 2.30 | 0.09 | 0.95 | 0.07 | 0.22 |

Bolded values indicate tests with P-values below the alpha value of 0.05 to indicate significance. Pairwise tests were conducted with the paired Wilcoxon signed rank test with a Bonferroni multiple test correction.

**Supplementary Table S3** Differential cell fraction statistical test results with IDOL and extended blood reference.

| **Cell Type** | **% at Baseline** | **% at Malaria** | **% at Recovery** | **Friedman P** | **Baseline vs Recovery P** | **Baseline vs Malaria P** | **Malaria vs Recovery P** |
| --- | --- | --- | --- | --- | --- | --- | --- |
| Monocytes | 7.04 | 12.03 | 6.10 | 1.00 | 1.00 | 1.00 | 0.44 |
| **NK cells** | **9.04** | **5.60** | **7.69** | **0.01** | **0.26** | **0.02** | **0.16** |
| Memory B cells | 6.98 | 7.59 | 6.15 | 0.22 | 0.18 | 0.40 | 0.18 |
| Naïve B cells | 11.46 | 9.86 | 11.26 | 0.20 | 0.84 | 0.24 | 0.84 |
| Memory CD4 T | 10.20 | 9.64 | 11.18 | 0.08 | 0.47 | 0.83 | 0.44 |
| Naïve CD4 T | 23.31 | 20.70 | 24.63 | 0.66 | 0.64 | 0.64 | 0.64 |
| **Memory CD8 T*** | **10.40** | **4.55** | **8.51** | **0.01** | **0.25** | **0.02** | **0.06** |
| Naïve CD8 T | 7.90 | 8.40 | 8.45 | 0.21 | 0.80 | 0.80 | 0.80 |
| Regulatory T | 3.85 | 3.91 | 4.24 | 0.61 | 0.60 | 0.95 | 0.60 |
| **Neutrophils** | **2.78** | **10.38** | **4.48** | **0.00** | **0.38** | **0.02** | **0.04** |
| Eosinophils | 0.00 | 0.00 | 0.00 | NA | NA | NA | NA |
| Basophils | 0.00 | 0.46 | 0.25 | 0.06 | 0.26 | 0.17 | 0.34 |

Bolded values indicate tests with P-values below the alpha value of 0.05 to indicate significance. Pairwise tests were conducted with the paired Wilcoxon signed rank test with a Bonferroni multiple test correction. *The total CD8 T cell count was also statistically significant (pval < 0.05) by Friedman test in the DNA-methylation derived estimates.

**Supplementary Table S4** Between-deconvolution proportion correlation analysis.

| **Cell Type** | **Pearson** | **P-value** | **lm Intercept** | **lm Slope** | **Adjusted R^2^** |
| --- | --- | --- | --- | --- | --- |
| Monocytes | 0.96 | 0.00 | -0.89 | 0.69 | 0.92 |
| Naïve CD4 T | 0.82 | 0.00 | 13.15 | 0.94 | 0.66 |
| CD8 T | 0.60 | 0.00 | 9.95 | 0.59 | 0.33 |
| Naïve B | 0.82 | 0.00 | -2.71 | 0.67 | 0.66 |
| NK cells | 0.78 | 0.00 | -1.72 | 0.59 | 0.59 |
| Memory CD4 T | 0.27 | 0.20 | 5.77 | 0.24 | 0.03 |
| Neutrophils | 0.34 | 0.10 | 5.15 | 13.79 | 0.08 |
| Memory B | 0.30 | 0.15 | 6.56 | 0.46 | 0.05 |
| Eosinophils | 0.00 | 1.00 | 0.00 | 0.00 | 0.00 |
| Regulatory T | 0.27 | 0.20 | 3.81 | 0.34 | 0.03 |

These are the Pearson correlation values, corresponding p-values, and linear modeling results for all the cell types that were compared. "lm" is the abbreviation for linear model.

**Supplementary Table S5** Summary of deconvolution-sensitive DEGs

| **Cell-type adjustment** | **Contrast** | **No. of deconvolution-sensitive DEGS** |
| --- | --- | --- |
| CD8 T | Baseline vs Malaria | 18 |
| CD8 T | Malaria vs Recovery | 13 |
| Prin. Comp. | Baseline vs Malaria | 5 |
| Prin. Comp. | Malaria vs Recovery | 5 |
| Naïve CD4 T | Malaria vs Recovery | 3 |
| Naïve CD4 T | Baseline vs Malaria | 2 |
| NK cell | Baseline vs Malaria | 1 |
| Naïve B | Malaria vs Recovery | 1 |
| Memory CD4 T | Malaria vs Recovery | 1 |

The number of deconvolution-sensitive DEGs are counted by contrast and cell-type adjustment. Deconvolution-sensitive DEGs are those that fall in the outer 0.3% of the distribution of orthogonal distances from the identity line in Figure 4. "Prin. Comp." refers to the principal components adjusted modeling results. More information about individual DEGs is available in Supplementary Dataset 1.

**Supplementary Table S6** Summary of deconvolution-sensitive DMPs

| **Cell-type adjustment** | **Contrast** | **No. of deconvolution-sensitive DMPS** |
| --- | --- | --- |
| CD8 T | Baseline vs Malaria | 45 |
| NK cells | Baseline vs Malaria | 14 |
| CD8 T | Malaria vs Recovery | 14 |
| Prin. Comp. | Baseline vs Malaria | 4 |
| Prin. Comp. | Malaria vs Recovery | 4 |
| Memory CD4 T | Baseline vs Malaria | 2 |
| Memory CD4 T | Malaria vs Recovery | 2 |

The number of deconvolution-sensitive DMPs are counted by contrast and cell-type adjustment. Deconvolution-sensitive DMPs are those that fall in the outer 0.3% of the distribution of orthogonal distances from the identity line in Figure 6. More information about individual DMPs is available in Supplementary Dataset 2.

**Supplementary Figure S1.** Cell proportion estimates for cell types not included on Figure 2a.


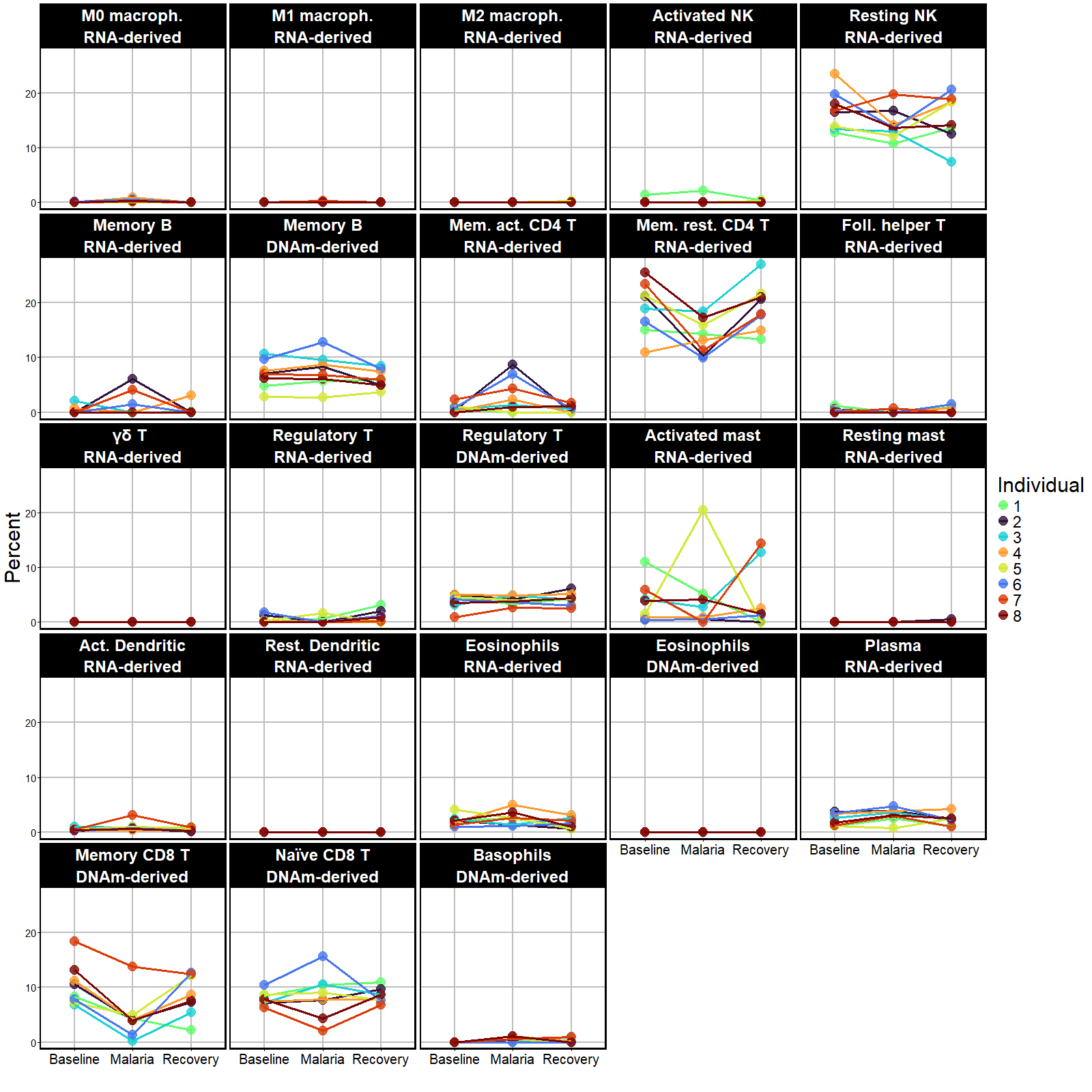


Each panel in this plot represents the percent estimates that are transcriptome-derived (panels labeled with "RNA-derived") and the DNA-methylation-derived (panels labeled with "DNAm-derived") for each cell type that is not included on Figure 2a. Each individual is represented by a color and labeled on the legend on the right side of the figure. Each panel shows the Baseline, Malaria, and Recovery time points for each child. "Baseline" is from when the child was asymptomatic and *plasmodium*-negative on blood smear and the first collected sample. "Malaria" is during the subsequent acute, febrile malaria illness when the child was brought to the clinic for treatment. Lastly, "Recovery" is collected ~6 weeks after the malaria illness and the child has become asymptomatic, afebrile, and *plasmodium*-negative on blood smear.

**Supplementary Figure S2.** Loading vectors for top two principal components in transcriptome-derived and DNAm-derived analyses.


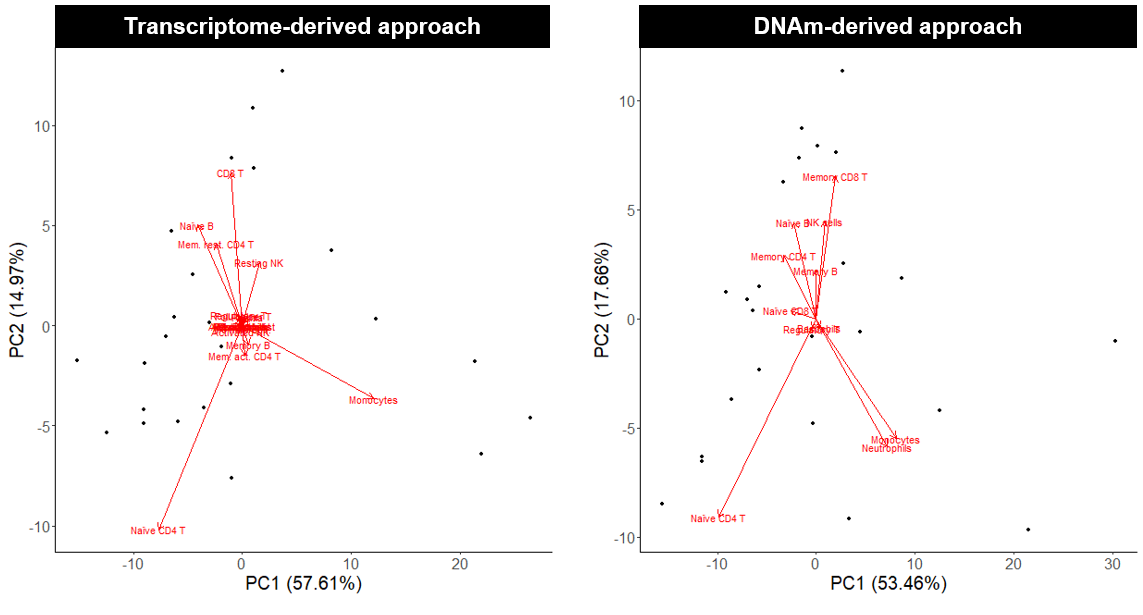


These panels represent the top contributing cell types that drive cell-type variation as calculated with transcriptome-derived (left) and DNA-methylation-derived (right) deconvolution approaches. The arrows demonstrate the influence of each cell type on the final PC positioning based on a singular value decomposition algorithm in the R package prcomp(). Each arrow is labeled with the contributing cell type, the length of the arrow corresponds to the magnitude of effect and the direction shows the relationship that cell type has with other cell types in the rotated Eigen space.
